# Deep Learning Algorithms with Demographic Information Help to Detect Tuberculosis in Chest Radiographs in Annual Workers’ Health Examination Data

**DOI:** 10.3390/ijerph16020250

**Published:** 2019-01-16

**Authors:** Seok-Jae Heo, Yangwook Kim, Sehyun Yun, Sung-Shil Lim, Jihyun Kim, Chung-Mo Nam, Eun-Cheol Park, Inkyung Jung, Jin-Ha Yoon

**Affiliations:** 1 Department of Biostatistics and Computing, Yonsei University Graduate School, Seoul 03722, Korea; lbthinking91@gmail.com (S.-J.H.); cmnam@yuhs.ac (C.-M.N.); 2 The Institute for Occupational Health, Yonsei University College of Medicine, Seoul 03722, Korea; nor5teo23@yuhs.ac (Y.K.); yunsehyun@yuhs.ac (S.Y.); lssmail@yuhs.ac (S.-S.L.); jihyun0924@yuhs.ac (J.K.); 3 Division of Biostatistics, Department of Biomedical Systems Informatics, Yonsei University College of Medicine, Seoul 03722, Korea; 4 Department of Preventive Medicine, Yonsei University College of Medicine, Seoul 03722, Korea; ecpark@yuhs.ac

**Keywords:** deep learning, image, computer-assisted diagnosis, tuberculosis, convolutional neural network

## Abstract

We aimed to use deep learning to detect tuberculosis in chest radiographs in annual workers’ health examination data and compare the performances of convolutional neural networks (CNNs) based on images only (I-CNN) and CNNs including demographic variables (D-CNN). The I-CNN and D-CNN models were trained on 1000 chest X-ray images, both positive and negative, for tuberculosis. Feature extraction was conducted using VGG19, InceptionV3, ResNet50, DenseNet121, and InceptionResNetV2. Age, weight, height, and gender were recorded as demographic variables. The area under the receiver operating characteristic (ROC) curve (AUC) was calculated for model comparison. The AUC values of the D-CNN models were greater than that of I-CNN. The AUC values for VGG19 increased by 0.0144 (0.957 to 0.9714) in the training set, and by 0.0138 (0.9075 to 0.9213) in the test set (both *p* < 0.05). The D-CNN models show greater sensitivity than I-CNN models (0.815 vs. 0.775, respectively) at the same cut-off point for the same specificity of 0.962. The sensitivity of D-CNN does not attenuate as much as that of I-CNN, even when specificity is increased by cut-off points. Conclusion: Our results indicate that machine learning can facilitate the detection of tuberculosis in chest X-rays, and demographic factors can improve this process.

## 1. Introduction

X-rays represent the most basic form of radiography, and are often considered the first step in medical examinations of organs and structures surrounding the chest [1]. Chest X-rays may provide insight into the patient’s condition, as certain diseases are associated with heart and lung abnormalities. However, in certain situations, physicians other than radiologists may have difficulty making accurate diagnoses based solely on images. Hence, for almost 60 years, researchers have devoted substantial effort to developing methods for computer-aided diagnosis (CAD) [2].

Recently, research regarding convolutional neural networks (CNNs) for CAD has expanded to include chest X-rays, computed tomography (CT), and high-resolution CT (HR-CT) [3]. Furthermore, several studies have investigated the application of CAD based on magnetic resonance imaging (MRI) and functional MRI, as well as ultrasound images, infrared thermography, electroencephalography (EEG), digital histopathology, and endoscopy photographs [4,5,6,7]. Previous research has demonstrated that CAD is useful for diagnosing [8] and characterizing patterns [9] of tuberculosis, with decent diagnostic accuracy. While HR-CT provides more information than simple chest X-rays and exhibits good precision in patients with lung diseases [10], it is associated with relatively high doses of radiation exposure. Hence, among the various methods for CAD, simple chest X-rays may be the most appropriate for improving diagnostic performance and reducing patient exposure to radiation. 

Radiologists consider demographic variables important when interpreting chest X-rays, as these variables may influence the detection of disease patterns via deep learning methods. Although images can be used to extract certain demographic variables, and deep learning methods can be used to determine gender based on X-ray images [11], few studies have attempted to include demographic variables within CNNs. Thus, it remains unclear whether images alone are sufficient for decision-making, or whether the addition of any demographic variables to the CNN would increase the performance of CAD. 

In the present study, we aimed to test the performance of CNNs on detecting tuberculosis and evaluate the difference in performance between a CNN based on images only (I-CNN) and a CNN that includes demographic variables (D-CNN) for the classification of tuberculosis in chest X-ray images. We hope this study will help prevent tuberculosis among workers. 

## 2. Materials and Methods

### 2.1. Ethics Statement

Private records for all participants were anonymized prior to analysis. The Institutional Review Board (IRB) of Yonsei University Hospital, South Korea, approved this study (IRB number: Y-2017-0071).

### 2.2. Cohort Data and Definitions

In Korea, Article 43 of the Occupational Safety and Health Act specifies that workers must undergo both general and specialized medical examinations each year [12]. Furthermore, shift workers and those exposed to harmful substances must undergo specialized health examinations every six to 24 months, depending on the hazardous substances. For the present study, we utilized annual medical surveillance data for workers at Yonsei University, beginning in 2009. For this cohort, we collected information regarding demographic and clinical characteristics, as well as medical test results, including chest X-rays. Demographic characteristics included age, gender, height, weight, waist circumference, and body mass index. Clinical characteristics included visual acuity; hearing ability; blood pressure; protein urea; a complete blood count; lipid profile; cholesterol profile; level of hepatitis B antigens/antibodies; and levels of lead, mercury, cadmium, styrene, toluene, dimethyl formamide, benzene, etc. Medical data included chest X-rays, pulmonary function test results, audiogram results, and Pittsburgh Sleep Quality Index (PSQI) values for shift workers.

### 2.3. Tuberculosis Definition

Diagnoses of tuberculosis in chest X-rays were defined by individual radiologists acting in accordance with the Quality Assurance Program enforced by the Occupational Safety and Health Act [12]. Each diagnosis/interpretation was based on guidelines detailed in the Framework Act on Health Examinations [13]. Interpretations were categorized as follows: A (normal), B (recommend re-examination), C (calcifications and fibrosis), D-A (tuberculosis-mild), D-B (tuberculosis-moderate), D-C (tuberculosis-severe), E (tuberculosis-suspicious, requiring sputum analysis/further evaluation), F (non-tuberculosis diseases), and G (undefined). In the current study, categories D-A, D-B, D-C, and E were considered to reflect signs of tuberculosis in chest X-rays. 

### 2.4. Preprocessing: Lung Segmentation

**Figure 1:**
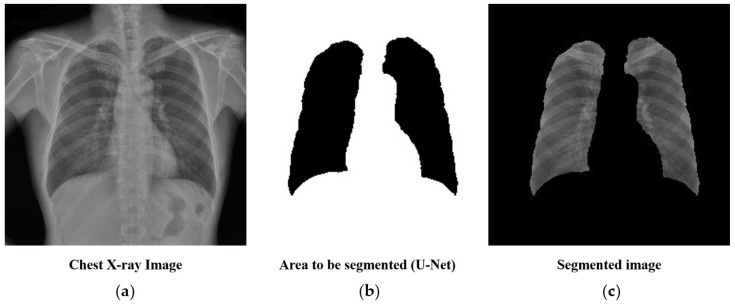
Lung segmentation using U-Net before training the convolutional neural network: (**a**) the original chest X-ray image, (**b**) a mask of lung structures segmented through U-Net, and (**c**) the final segmented image of the lungs.

Chest X-rays often include structures other than lungs, such as the spine and heart. As these areas are not useful for, and may hinder, the prediction of tuberculosis, we generated a mask containing lung structures only using U-Net [14]—a deep learning algorithm for biomedical image segmentation. To train the U-Net, we used 140 chest X-ray images with masks for lung parts. The trained U-Net achieved an average Dice coefficient of 0.9621 for 60 validation sets. The subsequent analysis was performed by cropping only the parts of the chest X-ray corresponding to the mask, as shown in Figure 1.

### 2.5. Deep Learning Methods

In the present study, CNNs were used for tuberculosis classification. CNNs are deep learning algorithms that generally extract image features via convolution and by pooling layers. Subsequently, the images were classified based on the features extracted. Several recent studies have demonstrated the remarkable performance of CNNs for the classification of medical images [8,15,16,17,18]. Furthermore, CNNs have been utilized in previous studies to establish CAD systems for disease diagnosis [19]. Among the available alternatives, we included the VGG19 [20], InceptionV3 [21], ResNet50 [22], DenseNet121 [23], and InceptionResNetV2 [24] CNN models in the present analysis. For our analysis, we used models that had been pre-trained using 1.2 million images from ImageNet (1000 categories) [25]. 

The total dataset included data for 39,677 individuals, and tuberculosis was found in a total of 1202 images. Among the total dataset, we randomly selected 1000 tuberculosis and 1000 non-tuberculosis patients to train CNN models. The remaining data were used to test both CNN models after training. All image resolutions were 2688 × 2688 pixels.

**Figure 2:**
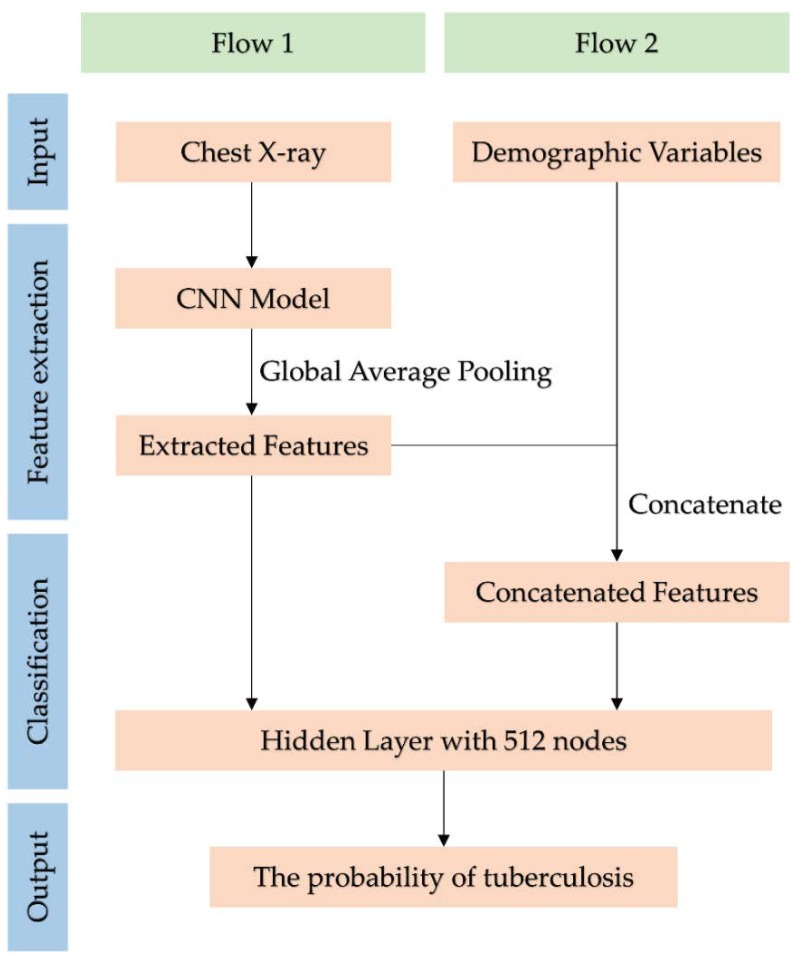
Flowchart of tuberculosis classification using the convolutional neural network (CNN) model. Flow 1 uses only chest X-rays for tuberculosis classification. Flow 2 uses demographic variables as well as chest X-rays.

In the present study, we included only four demographic variables: gender, age, height, and weight. The demographic characteristics of the training and test datasets are shown in Table 1. Convolution and layer pooling were utilized for existing CNN models, although global average pooling (GAP) was used instead of fully connected (FC) layers. Later, a hidden layer with 512 nodes was added for tuberculosis classification. In these modified CNN models, we combined the demographic variables with features extracted from the GAP, as shown in Figure 2. We then evaluated whether adding demographic variables improves the performance of tuberculosis classification for several CNN models. To avoid overfitting, 20% of the training dataset was randomly selected for validation. The test dataset was then evaluated using each trained CNN model. Model evaluation was based on significant differences in the area under the receiver operating characteristic (ROC) curve (AUC), following the addition of demographic variables.

We resized original images to 256 × 256 pixels for training CNN models. We set the batch size at 16 and the optimizer to stochastic gradient descent (SGD) while applying Nesterov momentum. The SGD learning rate, momentum, and decay were set to 0.001, 0.9, and 1 × 10^−6^, respectively. Data augmentation was performed for horizontal flips, vertical flips, rotations with a range of 30 degrees, width, and height shift with a range of 20%. Image pixel values and demographic variables were scaled such that they ranged between 0 and 1.

### 2.6. Statistical Analysis

Different characteristics between the training and test data sets were analyzed by a t-test and Pearson’s chi-squared test on age, gender, height, and weight. Comparisons of the AUC among models were analyzed by the non-parametric approach of DeLong [26] using the R packages of pROC [27]. A *p*-Value below 0.05 was regarded as a statistically significant level. All analyses of deep learning were performed using Python Version 3.6.3 (Python Software Foundation). The Python libraries numpy, pandas, scikit learn, OpenCV, Tensorflow, and Keras were used. 

## 3. Results

### 3.1. Basic Characteristics

In the training set, we observed significant differences in demographic characteristics, including age, gender, and weight, between tuberculosis-positive and tuberculosis-negative images (*p* < 0.05). Similar results were obtained in the test set (*p* < 0.05). Briefly, patients with tuberculosis-positive images tended to be older, were more likely to be male, and exhibited lower weight than those with tuberculosis-negative images (Table 1). 

### 3.2. Image only Convolutional Neural Networks Model Performance

The area under the curve values for VGG19, InceptionV3, ResNet50, and DenseNet121 were 0.957, 0.9523, 0.9219, 0.9315, and 0.9482, respectively, in the training set; and 0.9075, 0.8821, 0.8780, 0.8605, and 0.8851, respectively, in the test set (Table 2). 

Next, we compared AUC values for a CNN model containing only one demographic variable. In this case, we only used VGG19, which exhibited the best performance among the I-CNN models analyzed. The demographic variables with the second-highest and highest AUC values were then added to the model (Table 3). The AUC value increased by the greatest amount (by 0.0047) when weight was included as a factor, followed by age (by 0.0036) and gender (by 0.0032). However, when only one demographic variable was added, no significant differences in AUC values were observed (*p* > 0.05). The D-CNN model, which included both age and weight, resulted in an AUC increase of 0.0137 when compared with the I-CNN model. The *p*-value of the AUC difference was more significant than that obtained when using all demographic variables (*p* = 0.039 and 0.049, respectively). After the further addition of weight, age, and gender as factors in the I-CNN model, the AUC value increased by 0.0132 (*p* = 0.023).

**Figure 3:**
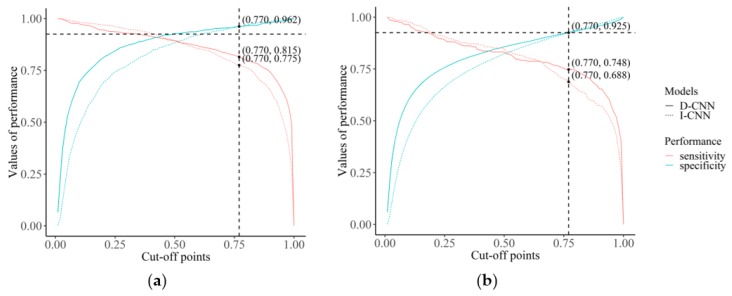
Value of sensitivity and specificity changed according to the cut-off point: (**a**) the sensitivity and specificity for the training data set and (**b**) the sensitivity and specificity for the test data set.

A further analysis was conducted to look at the difference in change of sensitivity of I-CNN and D-CNN if the value for the pre-selected cut-off point was changed. As shown in Figure 3, a specific cut-off point was selected on the point of intersection of specificity curves of I-CNN and D-CNN, which is around a cut-off value of 0.770. In contrast to specificity, sensitivity of D-CNN is greater than that of I-CNN (0.815 vs. 0.775, respectively). In the test data set, D-CNN shows greater sensitivity than I-CNN at the same cut-off point for the same specificity. Sensitivity of D-CNN does not attenuate as much as that of I-CNN even when specificity is increased by cut-off points.

## 4. Discussion 

In the present study, CNNs helped detect tuberculosis in workers’ heath examination data. We also evaluated the difference in performance when classifying tuberculosis between a CNN based only on images and a CNN that included demographic variables. For all CNN models except ResNet50 and InceptionResNetV2, the AUC differences between I-CNN and D-CNN models were statistically significant, indicating that demographic variables may be helpful for training CNN models and increasing performance with regard to tuberculosis classification. Furthermore, D-CNNs are more stable when the cut-off point is changed to increase the specificity or sensitivity of models. 

In general, CNN models for image classification are trained using images only. However, the distribution of demographic variables such as age and gender may vary from class to class. As shown in Table 1, the distribution of demographic variables also differed between participants with tuberculosis-positive and tuberculosis-negative images in the present study. We suspect that such differences may cause confounding effects in deep learning as well as in general statistical analysis. We attempted to adjust for these confounding effects by including both chest X-rays and demographic variables in the training dataset for CNN models (D-CNN). The AUC values for the test dataset increased for all CNN models after the addition of demographic variables. The VGG19, InceptionV3, and DenseNet121 models exhibited AUC improvements of up to 2.88% when demographic variables were included (Table 2, test dataset for DenseNet121). We further evaluated the performance of CNN models by adding one demographic variable at a time, hypothesizing that the inclusion of a greater number of demographic variables would result in even greater improvements in performance (Table 3). Our results suggest that training the CNN models using multiple demographic variables as well as chest X-rays significantly improves performance. 

X-rays provide minimal information when compared with other forms of imaging such as positron emission tomography (PET)/CT, which can provide more advanced information regarding the functional activity of an organ. Although PET/CT provides the most detailed radiographic information, assessment is associated with significant increases in radiation exposure [28]. While chest X-rays may provide less useful information than PET/CT, their use is more frequent due to the lower dose of radiation exposure. Recent advances in machine learning, especially with regard to deep learning, have improved the identification and classification of various diseases based on chest X-rays. Such improvements have both directly and indirectly enabled the extraction of more information from chest X-rays while minimizing the need for additional radiographic examination [29]. Hence, our results indicate that CNN models that include demographic variables can help prevent unnecessary radiation exposure and aid clinicians in extracting more information from medical images. 

In the current study, tuberculosis was defined based on the radiologists’ interpretations, which were made in accordance with established guidelines. However, additional steps are required to confirm diagnoses of tuberculosis. Firstly, patients typically experience excessive coughing for several weeks, following which physicians may simply prescribe medication for upper respiratory infection. In patients with severe or persistent symptoms, the physician may suspect tuberculosis and request a chest X-ray for differential diagnosis. Nonetheless, further evaluation (e.g., sputum analysis) is required to confirm the disease. Because such evaluation is important for patients with suspicious chest X-ray findings, our findings suggest that initial screening for both tuberculosis and suspicions of tuberculosis can aid in the management of tuberculosis symptoms.

The current study has several clinical implications. The process for confirming tuberculosis may take anywhere from several weeks to months. Given the extensive effort required in identifying tuberculosis, physicians may be unable to focus on actual treatment of patients with the disease. Our findings support the notion that CAD methods can be used to simplify the diagnostic process and improve disease management. While tuberculosis diagnoses are traditionally confirmed by a single doctor, allowing for the possibility of error, CAD methods can be regarded as a two-way confirmation system. In this case, the artificial intelligence system provides a diagnosis based on chest X-ray images, which can then be confirmed by the attending physician, drastically minimizing both human and machine error. Our results suggest that CAD methods can be used to improve diagnosis relative to traditional methods, which may improve the quality of treatment. 

The present study possesses some limitations of note, including the relatively low number of demographic features compared to the number of feature maps extracted by the CNN. For VGG19, the number of feature maps in the final layer of the feature extraction scheme was 512, while only four demographic features were utilized. When the features are fed into the global average pooling layer, a weight for each feature is assigned accordingly through back propagation. However, such unevenness in the number of features may have led to bias in the weight assignment. Our study is also limited by the resolution of the images: we utilized 256 × 256 down-sampled images due to limitations in computational power. Although higher image resolutions may improve results by providing greater detail, previous studies have indicated that this resolution is sufficient for CNN models for tuberculosis diagnosis [8]. In that study, the maximal accuracy was 0.99 (0.96–1.00), and it used the same 256 × 256 resolution. In our study, we used training and test datasets with different proportions of participants with tuberculosis-positive images. Balancing the class ratio of datasets for training the CNN models had a significant impact on performance [30]. Thus, for the training dataset, we extracted information for 1000 patients with positive or negative tuberculosis findings at the same ratio. The remaining data were used for the test dataset, in which 202 of 37,677 individuals were positive for tuberculosis (0.5%). Thus, we considered the test dataset appropriate for our assessment, as the proportion of individuals with positive findings was similar to the prevalence rate, and the sample was sufficiently large.

In conclusion, the results of the present study show that CNNs can help detect tuberculosis in chest X-rays, and highlight that demographic factors may improve the diagnosis of tuberculosis when included in such models. Although chest X-rays contain information regarding demographic characteristics, inclusion of this information in CNNs significantly improved prediction performance and model stability for specificity and sensitivity.

## Figures and Tables

**Table 1:** Summary of demographic variables for training and test datasets.

Variables	Training			Test		
	Tuberculosis		*p* Value *	Tuberculosis		*p* Value *
	Positive (*n* = 1000)	Negative (*n* = 1000)		Positive (*n* = 202)	Negative (*n* = 37,475)	
Age	50.08 ± 10.74	40.33 ± 11.07	<0.001	50.42 ± 10.48	40.30 ± 10.86	<0.001
Gender			<0.001			<0.001
Male	682 (68.20)	561 (56.10)		125 (61.88)	20,445 (54.56)	
Female	318 (31.80)	439 (43.90)		77 (38.12)	17,030 (45.44)	
Height	168.36 ± 8.33	167.85 ± 8.43	0.170	168.04 ± 8.53	167.54 ± 8.37	0.401
Weight	63.76 ± 11.42	64.98 ± 12.99	0.025	62.51 ± 10.74	64.43 ± 12.99	0.006

Values are presented as number (%) or mean ± standard deviation. * *p* value was calculated from *t*-test or chi-squared test.

**Table 2:** Comparison of the area under the curve (AUC) when using only images and when adding demographic variables.

Models	Training				Test			
	AUC			*p* Value	AUC			*p* Value
	I-CNN *	D-CNN **	Difference		I-CNN *	D-CNN **	Difference	
VGG19	0.9570	0.9714	0.0144	<0.001	0.9075	0.9213	0.0138	0.049
InceptionV3	0.9523	0.9616	0.0093	0.014	0.8821	0.9045	0.0224	0.033
ResNet50	0.9219	0.9250	0.0031	0.434	0.8780	0.8955	0.0175	0.051
DenseNet121	0.9315	0.9472	0.0157	0.002	0.8605	0.8893	0.0288	0.011
InceptionResNetV2	0.9482	0.9455	0.0027	0.407	0.8851	0.8864	0.0013	0.888

* I-CNN: convolutional neural network only using images; ** D-CNN: I-CNN with demographic variables added.

**Table 3:** AUC comparison of various demographic variable combinations for the training dataset: in reference to convolutional neural networks using I-CNN.

Input Variables	AUC	*p* Value *
I-CNN	0.9075	-
I-CNN + Gender	0.9107	0.657
I-CNN + Age	0.9111	0.602
I-CNN + Weight	0.9122	0.468
I-CNN + Height	0.9091	0.802
I-CNN + Weight + Age	0.9212	0.039
I-CNN + Weight + Age + Gender	0.9207	0.023
I-CNN + Weight + Age + Gender + Height	0.9213	0.049

* *p* value was calculated for the difference between the AUC based on I-CNN.
